# Efficacy and safety comparison of neoadjuvant chemotherapy followed by surgery and upfront surgery for treating intrahepatic cholangiocarcinoma: a systematic review and meta-analysis

**DOI:** 10.1186/s12876-023-02754-y

**Published:** 2023-04-12

**Authors:** Zijiao Yang, Xia Jiang

**Affiliations:** 1 West China School of Medicine, Sichuan University, Chengdu, 610000 China; 2 Regenerative Medicine Research Center, West China Hospital, Sichuan University, Chengdu, 610000 China

**Keywords:** Cholangiocarcinoma, Intrahepatic cholangiocarcinoma, Neoadjuvant chemotherapy, Surgery, Upfront surgery

## Abstract

**Background and aims:**

Currently, surgical resection is the most commonly performed and effective treatment for intrahepatic cholangiocarcinoma (ICC) worldwide. However, the prognosis of ICC is unsatisfactory. This study aimed to compare the efficacy and safety of neoadjuvant chemotherapy followed by surgery and upfront surgery in treating intrahepatic cholangiocarcinoma (ICC). The study also intends to explore whether chemotherapy should be introduced before surgery and which populations should be considered for neoadjuvant chemotherapy.

**Method:**

Four databases, including PubMed, EMBASE, Cochrane Library, and Web of Science, were searched from their inception dates to January 2022 for relevant articles. The statistical analysis was performed using the Review Manager Software (version5.3). The non-randomized interventions (ROBINS-I) was used to assess the methodological quality of included studies and the overall quality of evidence was assessed through the Grading of Recommendations Assessment, Development, and Evaluation (GRADE) tool. Moreover, the primary outcomes included 1-year, 3-year and 5-year overall survival (OS), while the secondary outcomes were R0 resection, 1-year, 3-year and 5-year recurrence-free survival (RFS), postoperative complications and ninety-day postoperative mortality.

**Results:**

Five studies involving 2412 patients were included in this meta-analysis. There was no significant difference in 1-year OS, 3-year OS, 1-year, 3-year and 5-year RFS, postoperative complications and ninety-day postoperative mortality between the two groups. However, the meta-analysis showed that the neoadjuvant chemotherapy group had a better 5-year OS benefit in ICC patients than the upfront surgery group (OR = 1.27, 95% CI: 1.02–1.58), while the R0 resection rate was lower in neoadjuvant chemotherapy group than that in the upfront surgery group (OR = 0.49, 95% CI: 0.26–0.91).

**Conclusion:**

Compared with the upfront surgery, neoadjuvant chemotherapy followed by surgery could prolong the 5-year OS without increasing the risk of postoperative complications in ICC patients. Considering that the patients in the neoadjuvant chemotherapy followed by surgery group had more advanced ICC cases, the benefits of neoadjuvant chemotherapy may be more significant in patients with more advanced ICC.

**Supplementary Information:**

The online version contains supplementary material available at 10.1186/s12876-023-02754-y.

## Introduction

Cholangiocarcinoma is a malignant tumor originating from bile duct epithelial cells and can be divided into intrahepatic cholangiocarcinoma (ICC) and extrahepatic cholangiocarcinoma, which can be classified into hilar and distal cholangiocarcinoma based on their anatomical location [1, 2]. ICC is the second most common liver tumor after hepatocellular carcinoma, accounting for 10% to 20% of all cholangiocarcinoma [3–5]. Surgical resection is the widely accepted and potentially curative therapy of choice for ICC, and the National Comprehensive Cancer Network (NCCN) guidelines recommend upfront surgery for resectable and non-metastatic ICC [6]. However, most ICC cases are usually advanced and unresectable, with multiple intrahepatic lesions and distant metastasis due to late diagnosis [7]. Although about 15% of ICC are resectable, the median survival is less than 3 years [4, 8]. Additionally, ICC is very prone to recurrence and metastasis after surgery, resulting in a relapse in about 22% of patients within six months after surgery [9], with lower 5-year overall survival (OS) (less than 40%) and 5-year recurrence-free survival (RFS) [10, 11]. Thus, ICC has a very poor prognosis.

The BILCAP phase III randomized controlled trial showed that using capecitabine as adjuvant chemotherapy following surgery can improve OS in patients with resected cholangiocarcinoma and gallbladder cancer [12]. The trial has promoted the widespread adoption of capecitabine as a clinical practice standard for adjuvant therapy and has been included in the treatment guidelines for biliary tract cancer, including the ASCO guidelines, in various countries [13, 14]. However, the treatment has been criticized for its ability to represent the true standard of care since postoperative capecitabine therapy failed to improve OS in the intention-to-treat population, which was the primary endpoint [15, 16]. Furthermore, two other phase-III randomized clinical trials also failed to show whether adjuvant chemotherapy based on gemcitabine [17] or gemcitabine plus oxaliplatin [18] improves the OS or RFS in patients with biliary tract cancer. Therefore, it can be concluded that not all patients can benefit from adjuvant therapy, whose effectiveness is closely related to the types of chemotherapy drugs. Most importantly, most ICC are unresectable, making it impossible for the patients to undergo postoperative adjuvant chemotherapy.

In such a treatment dilemma, neoadjuvant chemotherapy, which may be used for local de-escalation and systemic control of ICC, is an appealing approach. Recently, many clinicians have reported unexpected results from neoadjuvant chemotherapy followed by surgery as the treatment for unresectable ICC; however, these studies mostly represent case reports with varying chemotherapy regimens [19–29]. Two studies using propensity score matching analysis showed that surgical resection had similar postoperative outcomes and survival as that of liver transplantation in patients with ICC [30, 31]. Another study showed that neoadjuvant chemotherapy and/or chemoradiation could reduce the risk of death from resectable ICC by 23% compared with upfront surgery [32]. These results suggest that neoadjuvant chemotherapy followed by surgery could improve the prognosis of patients with ICC, especially the locally advanced ICC patients. However, there were also studies showing that negative margins (> 1 cm) rather than neoadjuvant therapy can increase the survival of patients with cholangiocarcinoma [33]. Moreover, considering the priority treatment mode of ICC surgery, some scholars question whether the introduction of neoadjuvant chemotherapy prolongs the waiting time for surgical resection (about 6.8 months) and whether the disease will be delayed or even worsened during this period [33, 34].

With such controversies and the lack of prospective studies, a systematic review and meta-analysis could provide a better understanding of these treatment regimens using the currently available research. This study compared the neoadjuvant chemotherapy followed by surgery with upfront surgery for ICC treatment using the latest and most comprehensive studies to obtain high quality evidence to guide their clinical application.

## Methods

This study was performed in accordance with the Preferred Reporting Items for Systematic Review and Meta-Analysis (PRISMA) guidelines [35].

### Search strategy

We searched PubMed, EMBASE, Cochrane Library, and Web of Science databases from their inception dates to January 2022 to obtain the relevant published articles. The search involved the use of MeSH terms and (or) free-text terms, including “Bile duct neoplasms,” “Biliary tract cancer*,” “Biliary tract cancer*,” “Cholangiocarcinoma,” “Intrahepatic cholangiocarcinoma*,” “Intrahepatic bile duct cancer,” “Neoadjuvant therapy,” “Neoadjuvant chemotherapy,” and “Preoperative chemotherapy.” The reference lists of the articles were also searched to obtain eligible related articles. The detailed search strategies of PubMed were presented in Supplementary Table  1.

### Inclusion criteria and exclusion criteria for this systematic review

The inclusion criteria for the articles were: (1) the study should be comparing neoadjuvant chemotherapy followed by surgery with upfront surgery in treating ICC; (2) the study should have reported at least one outcome of interest such as RFS, OS, R0 resection rate, complications, or mortality; (3) in case of duplication, only the most detailed and complete studies were included for data extraction; (4) the study should either be randomized controlled trials or non-randomized controlled trials. Articles published only in English were included.

Studies were excluded if: (1) patients did not suffer from ICC; (2) other treatments, such as liver transplantation, were used on ICC patients; (3) they were single-arm studies or case reports; (4) they had no original data included in the manuscript.

### Data extraction

Two reviewers (ZY and XJ) independently extracted the following data from the included studies: (1) general information including first author, publication year, country, study center, study design, interventions, sample size, and follow-up; (2) baseline patient characteristics such as age, sex, disease stage, chemotherapy regimens, RFS, OS, hospital stay duration, complications, and surgical margins; (3) results of the methodological quality evaluation and outcomes. Any disagreements were discussed and resolved by asking a third party.

### Bias risk assessment and assessment of certainty of evidence

The non-randomized interventions (ROBINS-I) [36] was used to assess the methodological quality of included studies, including confounding factors, selection of participants into the study, classification of interventions, deviations from intended interventions, missing data, measurement of outcomes, and selection of the reported results. The quality of evidence of the included studies was classified as high, moderate, low, or very low according to the Grading of Recommendations Assessment, Development, and Evaluation (GRADE) principles [37].

### Outcomes of interest

#### Primary outcomes

The primary outcomes included 1-year, 3-year, and 5-year OS.

#### Secondary outcomes

Secondary outcomes included R0 resection, 1-year, 3-year, and 5-year RFS, postoperative complications and ninety-day postoperative mortality.

### Statistical analysis

A meta-analysis was performed to compare the primary and secondary outcomes of interest between neoadjuvant chemotherapy followed by surgery and upfront surgery using the Review Manager 5.3. Dichotomous and continuous variables were presented as odds ratios (OR) and mean difference (MD), respectively, with a 95% confidence interval (CI). Moreover, the Mantel–Haenszel (MH) and inverse-variance (IV) methods were applied for dichotomous and continuous variables, respectively. Heterogeneity was assessed through the Chi-square (χ2) and heterogeneity (*I*2) test statistics, of which the latter could be divided into low (*I*2 < 25%), moderate (25 > *I*2 < 50%), and high heterogeneity (*I*2 > 50%) [38]. We used the fixed-effect model when the *I*2 value was < 50%; otherwise, a random-effects model was applied. The statistical significance of the *p* < 0.05 value was determined using the *Z* test. However, publication bias analysis was not performed because we included fewer studies (less than 10).

## Results

### Study selection

**Fig. 1:**
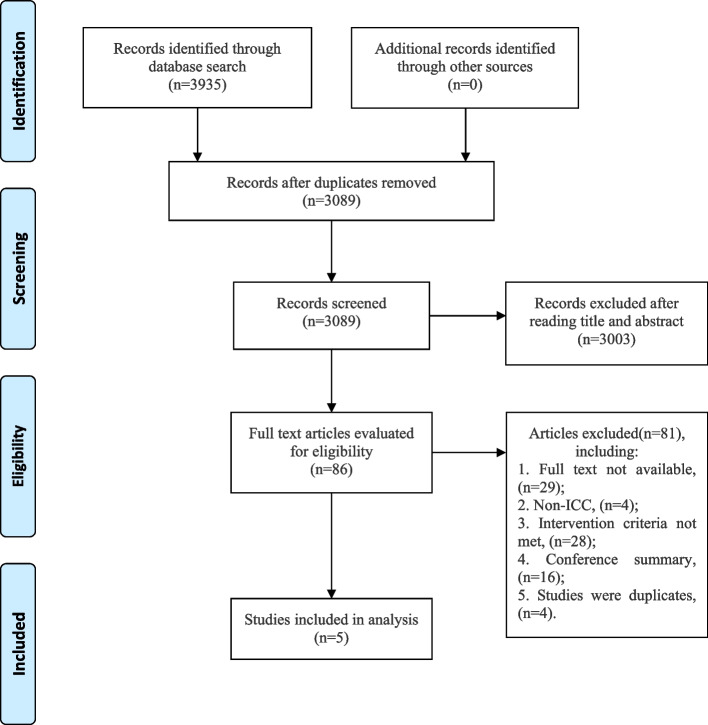
The PRISMA flow chart of the studies selection

We initially identified 3935 potentially relevant studies and retained 3089 for screening after removing the duplicated studies. Among these, 3003 studies were excluded after the title and abstract screening, and full text of 86 studies was read. We subsequently excluded 81 studies including 3 studies [39–41] that explored the effects of concurrent chemoradiotherapy in patients with biliary tract cancer and 1 study [42] did not provide sufficient original data in the manuscript on patients with advanced-stage. Eventually, 5 studies with 2412 patients were included in this meta-analysis. Of these, two studies were conducted in the USA [32, 43], two in France [44, 45], and one in the multicenter [46]. The PRISMA flow chart of the systematic literature search is presented in Fig. 1.

### Study characteristics and quality assessment

The baseline characteristics and types of all included studies are shown in Table 1. Among the 2412 patients included in the meta-analysis, 640 were under the neoadjuvant chemotherapy followed by surgery group, while 1772 were under the upfront surgery group. The characteristics and general information of all included studies are summarized in Table 1.

**Table 1:** Characteristics of the included studies

Author	Year	Study center	Type of study	Inclusion period	Treatment (Chemotherapy regimens)	No. of patients	Age (Mean)	Male sex (%)	Tumor AJCC stage	Number of tumors	Tumor size (cm)	Positive lymph node (%)	Lymphadenectomy	Vascular invasion (%)	Perineural invasion (%)	Hospital stay (Days)	Follow-up (Months)	Outcomes
Mason et al. [32]	2021	NCDB, USA	RS	2006 to 2016	Neoadjuvant chemotherapy followed by surgery (NA)	516	58.9	246 (47.7)	I–III	NA	≤ 5: 192; 6–10: 200; 11–15: 53; > 15: 20; Missing: 51	91 (17.6)	306 (59.3)	NA	NA	NA	NA	③④
					Upfront surgery	516	59.9	259 (50.2)	I–III	NA	≤ 5: 215; 6–10: 206; 11–15: 48; > 15: 15; Missing: 32	90 (17.4)	275 (53.3)	NA	NA	NA		
Sutton et al. [43]	2021	OHSU Knight Cancer Institute Liver Database, USA	RS	2004 to 2017	Neoadjuvant chemotherapy followed by surgery (Gem-Cis: 9; FOLFOX: 1)	10	58	3 (30)	I–III (I: 3, II: 3, III: 4)	Multiple tumors: 14; Single tumors: 38	5.45 (2.9–7.5)^a^	8 (15.4)	NA	19 (36.5)	14 (27)	8 (6–10)	27	①②③⑤⑥
					Upfront surgery	42	65	23 (54.8)	I–III (I: 17, II: 14, III: 11)				NA					
Le Roy et al. [44]	2018	Paul Brousse Hospital, France	RS	2000 to 2013	Neoadjuvant chemotherapy followed by surgery (mainly GEMOX; Other: Gem-based, Fluorouracil, Oxaliplatin and Irinotecan)	39	60	NA	Locally advanced	2.6 ± 3.3	75.0 (57–110)^a^	8 (20.5)	NA	6 of 35 (17.1)	3 of 14 (21)	14 (12–21)	93.3	①②③④⑤⑥⑦⑧⑨
					Upfront surgery	82	65	NA	Resectable	1.6 ± 1.8	70.0 (50–93)^a^	15 (18.3)	NA	10 of 80 (12.5)	17 of 42 (40)	12 (10–21)		
Riby et al. [45]	2020	Pontchaillou Hospital, France	RS	1997 to 2017	Neoadjuvant chemotherapy followed by surgery (Seven chemotherapy regimens, mainly GEMOX and Cis-based)	13	60	8 (61.5)	Initially unresectable	1 (1–10)^b^	7 (3–12)^b^	NA	NA	Macrovascular invasion: 0 (0)	5 (38.5)	NA	85.5	①②③④⑤⑥⑦⑧⑨
					Upfront surgery	137	67	100 (73)	Resectable	1 (1–10)^b^	6 (1–17)^b^	NA	NA	Macrovascular invasion: 14 (10.2)	30 (22.4)	NA		
Buettner et al. [46]	2017	Multicentre	RS	1990 to 2016	Neoadjuvant chemotherapy followed by surgery (Intra-arterial chemotherapy: 18; systemic chemotherapy: 44)	62	60	37 (59.7)	I: 7 (24.1), II: 6 (20.7), III: 6 (20.7), IV: 10 (34.5) (More advanced)	1 (1–1)	7.1 (5.0–10.2)	15 (24.2)	39 (70.9)	Major vascular invasion: 5 (8.9); Microvascular invasion: 25 (48.1)	15 (30.6)	9	27.6	①②③④⑤⑥⑦⑧⑨
					Upfront surgery	995	NA	NA	I: 282 (48.0), II: 160 (27.2), III: 22 (3.7), IV: 124 (21.0)	1 (1–1)	6.0 (4.2–8.8)	169 (17.0)	424 (43.7)	Major vascular invasion: 95 (9.8); Microvascular invasion: 232 (24.4)	137 (15.6)	12		

*NCDB* National Cancer Database, *OHSU* Oregon Health & Science University, *Gem* Gemcitabine, *Cis* Cisplatin, *FOLFOX* 5-Fluorouracil, Oxaliplatin, Folinic Acid, *GEMOX* Gemcitabine and Oxaliplatin, *NA* Not Available, *RS* Retrospective Study, *NOS* Newcastle–Ottawa Scale; Values are ^a^ median (IQR) and ^b^median (range)

①: 1-year OS; ②: 3-year OS; ③: 5-year OS; ④: R0 resection; ⑤: 1-year RFS; ⑥: 3-year RFS; ⑦: 5-year RFS; ⑧: Postoperative complications; ⑨: Ninety-day postoperative mortality

Two studies [32, 46] were classified as having a serious risk of bias, and the other three studies [43–45] were classified as having a moderate risk of bias. All of the above assessments were based on 5-year OS, the assessments based on all outcomes were shown in Table 2, and the quality of evidence of the included studies was shown in Table 3.

**Table 2:** Quality assessment of included studies using ROBINS-I

Reference	Outcomes	Bias due to confounding	Bias in selection of participants into the study	Bias in classification of Interventions	Bias due to deviations from intended interventions	Bias due to missing data	Bias in measurement of outcomes	Bias in selection of the reported results	Overall risk of bias
Mason et al. [32]	5-year OS	Moderate	Moderate	Low	Serious	Moderate	Low	No information	Serious
	R0 resection	Moderate	Moderate	Low	Serious	Moderate	Moderate	No information	Serious
Sutton et al. [43]	1-year OS	Moderate	Moderate	Low	Low	Moderate	Low	No information	Moderate
	3-year OS	Moderate	Moderate	Low	Low	Moderate	Low	No information	Moderate
	5-year OS	Moderate	Moderate	Low	Low	Moderate	Low	No information	Moderate
	1-year RFS	Moderate	Moderate	Low	Low	Moderate	Low	No information	Moderate
	3-year RFS	Moderate	Moderate	Low	Low	Moderate	Low	No information	Moderate
Le Roy et al. [44]	1-year OS	Moderate	Low	Low	Low	Low	Low	No information	Moderate
	3-year OS	Moderate	Low	Low	Low	Low	Low	No information	Moderate
	5-year OS	Moderate	Low	Low	Low	Low	Low	No information	Moderate
	R0 resection	Moderate	Low	Low	Low	Low	Moderate	No information	Moderate
	1-year RFS	Moderate	Low	Low	Low	Low	Low	No information	Moderate
	3-year RFS	Moderate	Low	Low	Low	Low	Low	No information	Moderate
	5-year RFS	Moderate	Low	Low	Low	Low	Low	No information	Moderate
	Postoperative complications	Moderate	Low	Low	Low	Low	Low	No information	Moderate
	Ninety-day postoperative mortality	Moderate	Low	Low	Low	Low	Low	No information	Moderate
Riby et al. [45]	1-year OS	Moderate	Low	Low	Low	Low	Low	No information	Moderate
	3-year OS	Moderate	Low	Low	Low	Low	Low	No information	Moderate
	5-year OS	Moderate	Low	Low	Low	Low	Low	No information	Moderate
	R0 resection	Moderate	Low	Low	Low	Low	Moderate	No information	Moderate
	1-year RFS	Moderate	Low	Low	Low	Low	Low	No information	Moderate
	3-year RFS	Moderate	Low	Low	Low	Low	Low	No information	Moderate
	5-year RFS	Moderate	Low	Low	Low	Low	Low	No information	Moderate
	Postoperative complications	Moderate	Low	Low	Low	Low	Low	No information	Moderate
	Ninety-day postoperative mortality	Moderate	Low	Low	Low	Low	Low	No information	Moderate
Buettner et al. [46]	1-year OS	Serious	Moderate	Low	Low	Moderate	Low	No information	Serious
	3-year OS	Serious	Moderate	Low	Low	Moderate	Low	No information	Serious
	5-year OS	Serious	Moderate	Low	Low	Moderate	Low	No information	Serious
	R0 resection	Serious	Moderate	Low	Low	Moderate	Moderate	No information	Serious
	1-year RFS	Serious	Moderate	Low	Low	Moderate	Low	No information	Serious
	3-year RFS	Serious	Moderate	Low	Low	Moderate	Low	No information	Serious
	5-year RFS	Serious	Moderate	Low	Low	Moderate	Low	No information	Serious
	Postoperative complications	Serious	Moderate	Low	Low	Moderate	Low	No information	Serious
	Ninety-day postoperative mortality	Serious	Moderate	Low	Low	Moderate	Low	No information	Serious

**Table 3:** Assessment of certainty of evidence according to GRADE for all outcomes

Certainty assessment						No of patients		OR (95% CI)	Certainty
Outcomes	**Risk of bias**	**Inconsistency**	**Indirectness**	**Imprecision**	**Publication bias**	**NC**	**US**		
1-year OS	Serious^a^	Not serious	Not serious	Not serious	Not assessed^c^	97/124	994/1256	0.94 (0.59–1.50)	⨁⨁⨁◯
3-year OS	Serious^a^	Not serious	Not serious	Not serious	Not assessed^c^	63/124	623/1256	1.17 (0.80–1.72)	⨁⨁⨁◯
5-year OS	Serious^a^	Not serious	Not serious	Not serious	Not assessed^c^	234/640	616/1772	1.27(1.02–1.58)	⨁⨁⨁◯
R0 resection	Serious^a^	Serious^b^	Not serious	Not serious	Not assessed^c^	432/630	1381/1730	0.49 (0.26–0.91)	⨁⨁◯◯
1-year RFS	Serious^a^	Not serious	Not serious	Not serious	Not assessed^c^	71/124	781/1256	0.95 (0.64–1.40)	⨁⨁⨁◯
3-year RFS	Serious^a^	Not serious	Not serious	Serious^c^	Not assessed^c^	47/124	539/1256	1.02 (0.68–1.52)	⨁⨁◯◯
5-year RFS	Serious^a^	Not serious	Not serious	Serious^c^	Not assessed^c^	34/114	476/1214	0.89 (0.57–1.39)	⨁⨁◯◯
Postoperative complications	Serious^a^	Not serious	Not serious	Serious^c^	Not assessed^c^	5/114	50/1214	1.23 (0.51–2.97)	⨁⨁◯◯
Ninety-day postoperative mortality	Serious^a^	Not serious	Not serious	Serious^c^	Not assessed^c^	5/114	50/1214	0.76 (0.28–2.01)	⨁⨁◯◯

*CI* Confidence interval, *NC* Neoadjuvant chemotherapy followed by surgery, *US* Upfront surgery

^a^High risk of bias

^b^High heterogeneity

^c^the sample size was smaller

### Meta-analysis

#### One-year OS

**Fig. 2:**
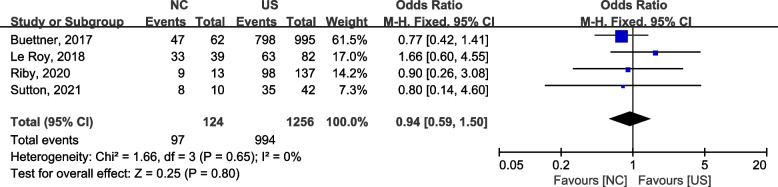
A forest plot of the 1-year OS between the neoadjuvant chemotherapy followed by surgery and upfront surgery for treating ICC

Four studies [43–46] reported 1-year OS of the two procedures, and a fixed effects model was used for the analysis because no significant heterogeneity was observed between the studies (2 = 1.66, *P* = 0.56,* I*2 = 0). We found no significant differences between the two groups (OR = 0.94, 95% CI: 0.59–1.50) (Fig. 2).

#### Three-year OS

**Fig. 3:**
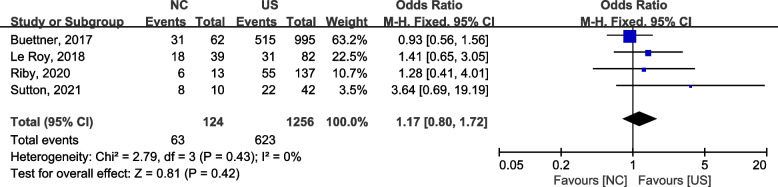
A forest plot for 3-year OS between the neoadjuvant chemotherapy followed by surgery and upfront surgery for treating ICC

Similarly, four studies [43–46] reported 3-year OS of the two procedures, and a fixed effects model was used for the analysis (2 = 2.79, *P* = 0.43,* I*2 = 0), which revealed that there was no significant difference between the two groups (OR = 1.17, 95% CI: 0.80–1.72) (Fig. 3).

#### Five-year OS

**Fig. 4:**
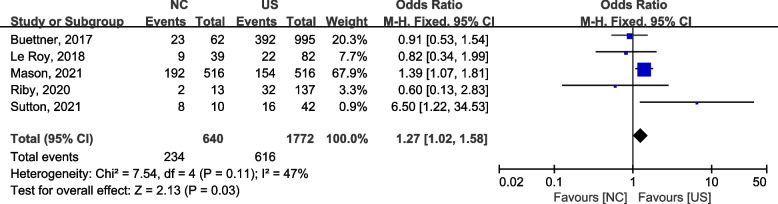
A forest plot for 5-year OS between the neoadjuvant chemotherapy followed by surgery and upfront surgery for treating ICC

All studies reported 5-year OS of the two procedures, and the meta-analysis revealed a statistically significant difference between the two groups (OR = 1.27, 95% CI: 1.02–1.58), with a moderate heterogeneity (2 = 7.54, *P* = 0.11,* I*2 = 47%) (Fig. 4).

#### R0 resection

Four studies [32, 44–46] reported R0 resection of the two procedures. We used a random effects model for the analysis because a high heterogeneity was observed between the studies (2 = 11.99, *P* = 0.007,* I*2 = 75%). The meta-analysis also showed a significant difference between the two groups (OR = 0.49, 95% CI: 0.26–0.91) (Table 4).

**Table 4:** Meta-analysis results of the secondary outcomes between the neoadjuvant chemotherapy followed by surgery and upfront surgery for treating ICC

Outcomes	No. of Studies	Assessment of Heterogeneity		No. of patients		Meta-analysis Results	
		*I*^2^ (%)	*P*	NC	US	OR ( 95% CI)	*P*
R0 resection	4 [32, 44–46]	75	0.007	630	1381	0.49 (0.26–0.91)	**0.02**
One-year RFS	4 [43–46]	0	0.41	124	1256	0.95 (0.64–1.40)	0.79
Three-year RFS	4 [43–46]	0	0.72	124	1256	1.02 (0.68–1.52)	0.94
Five-year RFS	3 [44–46]	0	0.70	114	1214	0.89 (0.57–1.39)	0.60
Postoperative complications	3 [48–50]	67	0.05	114	1214	1.23 (0.51–2.97)	0.65
Ninety-day postoperative mortality	3 [44–46]	0	0.76	114	1214	0.76(0.28–2.01)	0.58

*NC* Neoadjuvant chemotherapy followed by surgery, *US* Upfront surgery

#### One-year RFS

One-year RFS of the two procedures was reported by four studies [43–46]. The meta-analysis revealed no significant heterogeneity (2 = 2.88, *P* = 0.41,* I*2 = 0%) and difference (OR = 0.95, 95% CI: 0.64–1.40) between the two groups (Table 4).

#### Three-year RFS

Three-year RFS of the two procedures was also reported by four studies [43–46], and no significant difference between the two groups (OR = 1.02, 95% CI: 0.68–1.52) and heterogeneity (2 = 1.32, *P* = 0.72,* I*2 = 0%) between the studies was observed (Table 4).

#### Five-year RFS

Three studies [44–46] reported 5-year RFS of the two procedures, and our meta-analysis revealed no statistically significant difference between the two groups (OR = 0.89, 95% CI: 0.57–1.39) and heterogeneity between the studies (2 = 0.71, *P* = 0.70,* I*2 = 0) (Table 4).

#### Postoperative complications

Four studies [43–46] reported postoperative complications (Clavien-Dindo Grade ≥ III) of the two procedures, and the data from three studies [44–46] can be used for quantitative analysis. We used a random effects model for the analysis because a high heterogeneity was observed between the studies (2 = 6.00, *P* = 0.05,* I*2 = 67%). The meta-analysis showed there was no significant difference between the two groups (OR = 1.23, 95% CI: 0.51–2.97) (Table 4).

#### Ninety-day postoperative mortality

The ninety-day postoperative mortality of the two procedures was reported by three studies [44–46]. The meta-analysis revealed no statistically significant difference between the two groups (OR = 0.76, 95% CI: 0.28–2.01) and heterogeneity among the studies (2 = 0.54, *P* = 0.76,* I*2 = 0) (Table 4).

### Sensitivity analysis

Sensitivity analysis of the primary outcomes (1-year, 3-year, and 5-year OS, and R0 resection) and secondary outcomes (1-year, 3-year, and 5-year RFS, postoperative complications, and ninety-day postoperative mortality) was performed by removing one study at a time from the meta-analysis using the Review Manager 5.3 and testing their heterogeneity differences. The results indicated that, for R0 resection, the heterogeneity reduced significantly when the study by Mason et al. [32] was excluded (2 = 0.18, *P* = 0.92,* I*2 = 0); however, the recalculated results were consistent with those obtained when all studies were included. For postoperative complications, when the study by Buettner et al. [46] was excluded, the heterogeneity reduced significantly (*χ*2 = 0.32, *P* = 0.57, *I*2 = 0) while the recalculated results were consistent with those obtained when all studies were included (OR 0.74, 95% CI 0.35–1.55). However, the recalculated results showed that the postoperative complications in the upfront surgery group was fewer than the neoadjuvant chemotherapy followed by surgery group (OR 1.95, 95% CI 1.07–3.58) and the heterogeneity decreased (*χ*2 = 1.13, *P* = 0.29, *I*2 = 12%) when the study by Le Roy et al. [44] was excluded. No significant change was found in the overall statistical significance of the model.

## Discussion

Although the use of neoadjuvant chemotherapy in ICC treatment is still in the exploratory stage, its application in managing other cancer types has increased with promising results [47]. This is mainly due to the rarity of ICC, making randomized controlled trials and large prospective studies impractical [6]. Moreover, the lack of high-level evidence and the concerns about the toxic preoperative effects of chemotherapy drugs limit the use of neoadjuvant chemotherapy for ICC treatment. The delayed diagnosis, strong invasiveness and poor prognosis of ICC also make the existing treatment options insufficient, necessitating urgent development of effective interventions. Studies have shown that neoadjuvant chemotherapy is mainly used in ICC to downstage locally advanced tumors, improve R0 resection rate, prioritize or increase receipt of systemic treatment, and enhance patient selection for major surgery, thus, facilitating an in vivo effectiveness test of the treatment [7]. Therefore, it is important to determine whether neoadjuvant chemotherapy, particularly neoadjuvant chemotherapy followed by surgery, has an oncological advantage, such as survival benefits, to patients with ICC. If neoadjuvant therapy can only benefit some patients based on the individualized characteristics of the tumors, identifying such patients will greatly facilitate the future advancement and refinement of the treatment.

Our study found that the R0 resection rate was significantly lower in the neoadjuvant chemotherapy group than in the upfront surgery group. This may be due to the selection bias that patients in the neoadjuvant chemotherapy group were more likely to have more advanced or initially unresectable ICC across the five studies included in this analysis, while those in the upfront surgery group were resectable. This is also a common problem in non-randomized controlled trials, and although the authors tried to minimize the bias using propensity score matching analysis, it is difficult to eliminate the bias based on the current clinical criteria for choosing neoadjuvant chemotherapy for treating ICC [32, 46]. Previous studies have shown that R0 resection is an independent risk factor affecting the ICC prognosis in local control and long-term survival and is one of the outcomes pursued by surgeons [33, 39, 48]. However, although the short-term OS (1-year and 3-year OS) was not statistically different between the two groups, the 5-year OS was remarkably higher in the neoadjuvant chemotherapy group than in the upfront surgery group. A possible explanation could be that the introduction of neoadjuvant chemotherapy made the prognostic impact of R0 resection less important. That is, the positive prognostic impact of neoadjuvant chemotherapy outweighed the negative impact of R1 or R2 resection, consistent with our previous understanding of the vital role of R0 resection in malignancies treatment. Another possible explanation is that applying postoperative adjuvant chemotherapy and re-intervention when ICC recurs was not balanced between the two groups. Moreover, the overall postoperative adjuvant therapy is not widely used, and no quantitative comparison of ICC relapse re-intervention was found in the five studies. The large disparity in the number of patients between the two groups may have also contributed to the statistical Type I errors [49].

We also noted that the OS benefit of the neoadjuvant chemotherapy was only manifested on a long-term basis, which may be attributed to the tumor factors and recurrence pattern of ICC. Similarly, studies have shown no survival advantage when all ICC patients (stages I-III) are considered but recorded statistically significant differences in their five-year OS or median OS when only stage II-III patients were considered for analysis [6, 42]. Additionally, Marcus et al. [50] also found that for patients with more advanced disease, the receipt of neoadjuvant chemotherapy was associated with significantly improved survival compared to upfront surgery (Stage II, *P* = 0.040; Stage III, *P* = 0.003),while there was no statistically significant difference between the two treatment strategies in patients with clinical stage I (*P* = 0. 30). Thus, it could be inferred that neoadjuvant chemotherapy has better effects on patients with more advanced stage ICC, which are the most important stages in clinical practice. This is consistent with our finding that the neoadjuvant chemotherapy group had higher 5-year OS than the upfront surgery group despite the former having advanced-stage ICC participants. Sutton et al. also found that neoadjuvant chemotherapy was independently associated with improved 5-year OS when evaluating tumor stage management using multivariate analysis [43].

Although studies have shown that neoadjuvant therapy is an independent predictor of RFS [51], no statistical differences in short-term and long-term RFS were found between the two groups. This may be attributed to the high and early recurrence of ICC, which could not be inhibited by the neoadjuvant chemotherapy followed by surgery. Hu et al. retrospectively analyzed the recurrence patterns and timing of ICC after a curative-intent resection in 920 patients [52]. The study found that 66% of the patients experienced recurrence within a median follow-up in 38 months, with pure intrahepatic recurrence being the most common at 53.2%. Other studies also reported similar results showing that the most common recurrence site was the liver [9, 43–45]. Furthermore, in the event of recurrence, repeated surgical resection leads to better survival than other treatments [52]. Thus, the small number of patients included in the neoadjuvant chemotherapy group may have resulted in the absence of statistical significance in the difference between the RFS in this study. Large-sampled and high-quality studies are, therefore, needed to further validate and explore this phenomenon.

Clearly, not all patients with ICC could benefit from neoadjuvant therapy in terms of OS and RFS, especially the latter. This might have been due to the external confounding factor, which was the differences in the chemotherapy regimens used in the included studies, especially the choice of chemotherapy drugs. Two classic randomized controlled trials, ABC-02 and BT22, have demonstrated the efficacy and safety of cisplatin and gemcitabine in treating advanced biliary tract cancer [53]. Therefore, the neoadjuvant chemotherapy drugs in this study are mostly based on gemcitabine, and according to multiple case reports, gemcitabine administration seems like a very promising treatment in combination with other neoadjuvant chemotherapy drugs. However, there is no consensus on the choice of single or multiple agents in the neoadjuvant chemotherapy for ICC, and the dosage and chemotherapy cycle also vary widely among these reported studies. Although studies have shown no difference in the impact of single-drug or multi-drug treatment on the survival benefit of ICC patients [32, 54], clinicians seem to be more willing to try multiple agents therapy during neoadjuvant chemotherapy in ICC. Therefore, this necessitates a unified neoadjuvant chemotherapy regimen. Furthermore, the internal confounding factors are the individual differences among patients, such as tumor stage and genes, which may influence the patient’s response to neoadjuvant chemotherapy. Early identification of the patients potentially benefiting from neoadjuvant chemotherapy or prognosis prediction of the ICC patients may aid in providing personalized medical interventions. Accordingly, research on predictive models or tools has yielded initial results in this area [55–60].

With respect to safety, no statistical difference in severe postoperative complications [44, 61] and ninety-day postoperative mortality was found between the neoadjuvant chemotherapy followed by surgery and upfront surgery groups. Our findings are consistent with a study of Choi et al. [62] that based on the ACS-NSQIP database. In our study, four [43–46] studies reported Clavien-Dindo Grade ≥ III complications associated with the treatment, of which, one study [43] providing qualitative descriptions that neoadjuvant chemotherapy was not associated with Clavien-Dindo Grade ≥ III complications. The ninety-day postoperative mortality is an important outcome that is influenced by factors such as preoperative treatment, surgical quality, patients, surgeons, and medical institutions, and is a legitimate parameter that measures the safety of treatment procedures [63, 64]. Although these data are insufficient for evaluating the safety of neoadjuvant chemotherapy followed by surgery, the intraoperative, short-term and long-term complications after neoadjuvant chemotherapy need further studies.

In general, using neoadjuvant chemotherapy followed by surgery for treating ICC requires robust data for experimental evidence; however, this is limited by the lack of randomized controlled trials and the difficulty in developing large-scale studies. This study conducted a meta-analysis of the efficacy and safety of neoadjuvant chemotherapy using relevant articles published in recent years, and preliminary conclusions were drawn. The study also reviewed the results of the previous related studies. Despite all the findings, this study also has several limitations. First, we only included a few retrospective studies with fewer patients in the neoadjuvant chemotherapy group than in the upfront surgery group, which limits the quality of the study. Secondly, there were differences in the chemotherapy regimens used in the included studies, especially in selecting chemotherapy drugs, which is critical for studies involving drugs. Thirdly, the patients in the neoadjuvant group had more advanced ICC cases, resulting in a selection bias of patients because the bias could not be eliminated. Finally, the included studies reported very few short- and long-term complications necessary for detailed safety assessment of aspect treatments.

## Conclusions

Compared with the upfront surgery, neoadjuvant chemotherapy followed by surgery exhibited no significant RFS benefit, but it could prolong the 5-year OS of the ICC patients without increasing the risk of postoperative complications. Thus, neoadjuvant chemotherapy followed by surgery should be considered in ICC, especially in patients with more advanced disease.

## Supplementary Information

**Additional file 1: Supplementary Table 1.** The detailed search strategies of PubMed.

## Data Availability

All data generated or analyzed during this study are included in this published article and its supplementary information files.
