# Supplementary material for: Efficacy and safety comparison of neoadjuvant chemotherapy followed by surgery and upfront surgery for treating intrahepatic cholangiocarcinoma: a systematic review and meta-analysis
Source: BMC Gastroenterol. 2023 Apr 12;23:122. doi: 10.1186/s12876-023-02754-y (PMC10099833; doi:10.1186/s12876-023-02754-y)
Supplement: Supplementary file 1 — Additional file 1: Supplementary Table 1. The detailed search strategies of PubMed. [file 12876_2023_2754_MOESM1_ESM.docx]

|  |  | **Search Strategies** |
| --- | --- | --- |
| **P** | #1 | "Bile Duct Neoplasms"[Mesh] |
|  | #2 | (((((((((Bile Duct Neoplasm*[Title/Abstract]) OR (Bile Duct Cancer*[Title/Abstract])) OR (Cancer of the Bile Duct[Title/Abstract])) OR (Cancer of Bile Duct[Title/Abstract])) OR (Biliary Tract Neoplasm[Title/Abstract])) OR (Biliary Tract Cancer*[Title/Abstract])) OR (Cancer of Biliary Tract[Title/Abstract])) OR (Cancer of the Biliary Tract[Title/Abstract])) OR (biliary cancer[Title/Abstract])) OR (choledochus cancer[Title/Abstract]) |
|  | #3 | "Cholangiocarcinoma"[Mesh] |
|  | #4 | ((((((((((((((((((((Cholangiocarcinoma*[Title/Abstract]) OR (Cholangiocellular Carcinoma*[Title/Abstract])) OR (Extrahepatic Cholangiocarcinoma*[Title/Abstract])) OR (Intrahepatic Cholangiocarcinoma*[Title/Abstract])) OR (intrahepatic bile duct cancer[Title/Abstract])) OR (bile tract carcinoma[Title/Abstract])) OR (biliary carcinoma[Title/Abstract])) OR (biliary duct carcinoma[Title/Abstract])) OR (biliary tract carcinoma[Title/Abstract])) OR (cholangiolar carcinoma[Title/Abstract])) OR (extrahepatic bile duct carcinoma[Title/Abstract])) OR (extrahepatic biliary duct carcinoma[Title/Abstract])) OR (gall duct carcinoma[Title/Abstract])) OR (hilar cholangiocarcinoma[Title/Abstract])) OR (intrahepatic bile duct carcinoma[Title/Abstract])) OR (intrahepatic biliary duct carcinoma[Title/Abstract])) OR (malignant cholangioma[Title/Abstract])) OR (perihilar bile duct carcinoma[Title/Abstract])) OR (perihilar biliary duct carcinoma[Title/Abstract])) OR (perihilar cholangiocarcinoma[Title/Abstract])) OR (extrahepatic bile duct cancer[Title/Abstract]) |
|  | #5 | "Klatskin Tumor"[Mesh] |
|  | #6 | ((((((Klatskin Tumor[Title/Abstract]) OR (Klatskin's Tumor[Title/Abstract])) OR (Hilar Cholangiocarcinoma[Title/Abstract])) OR (Hilar Cholangiocarcinomas[Title/Abstract])) OR (Perihilar Cholangiocarcinoma[Title/Abstract])) OR (Perihilar Cholangiocarcinomas[Title/Abstract])) OR (distal cholangiocarcinoma[Title/Abstract]) |
|  | #7 | #1 OR #2 OR #3 OR #4 OR #5 OR #6 |
| **I** | #8 | "Neoadjuvant Therapy"[Mesh] |
|  | #9 | (((((((((((((((((((((((((((((((((((((((((Neoadjuvant Therapy[Title/Abstract]) OR (Neoadjuvant Therapies[Title/Abstract])) OR (Neoadjuvant Treatment*[Title/Abstract])) OR (Neoadjuvant Radiotherapy[Title/Abstract])) OR (Neoadjuvant Radiotherapies[Title/Abstract])) OR (Neoadjuvant Radiation Treatment*[Title/Abstract])) OR (Neoadjuvant Radiation Therapy[Title/Abstract])) OR (Neoadjuvant Radiation Therapies[Title/Abstract])) OR (Neoadjuvant Radiation*[Title/Abstract])) OR (Neoadjuvant Systemic Therapy[Title/Abstract])) OR (Neoadjuvant Systemic Therapies[Title/Abstract])) OR (Neoadjuvant Systemic Treatment*[Title/Abstract])) OR (Neoadjuvant Chemotherapy[Title/Abstract])) OR (Neoadjuvant Chemotherapies[Title/Abstract])) OR (Neoadjuvant Chemotherapy Treatment*[Title/Abstract])) OR (Neoadjuvant Chemoradiotherapy[Title/Abstract])) OR (Neoadjuvant Chemoradiotherapies[Title/Abstract])) OR (Neoadjuvant Chemoradiation Therapy[Title/Abstract])) OR (Neoadjuvant Chemoradiation Therapies[Title/Abstract])) OR (Neoadjuvant Chemoradiation Treatment*[Title/Abstract])) OR (Neoadjuvant Chemoradiation*[Title/Abstract])) OR (neo-adjuvant therapy[Title/Abstract])) OR (neo-adjuvant treatment[Title/Abstract])) OR (preoperative therapy[Title/Abstract])) OR (preoperative treatment[Title/Abstract])) OR (neo-adjuvant chemoradiation[Title/Abstract])) OR (neo-adjuvant chemoradiotherapy[Title/Abstract])) OR (preoperative chemoradiation[Title/Abstract])) OR (preoperative chemoradiotherapy[Title/Abstract])) OR (neo-adjuvant chemotherapy[Title/Abstract])) OR (preoperative chemotherapy[Title/Abstract])) OR (pre-operation chemotherapy[Title/Abstract])) OR (pre-operational chemotherapy[Title/Abstract])) OR (pre-operative chemotherapy[Title/Abstract])) OR (pre-operatory chemotherapy[Title/Abstract])) OR (pre-surgery chemotherapy[Title/Abstract])) OR (pre-surgical chemotherapy[Title/Abstract])) OR (preoperation chemotherapy[Title/Abstract])) OR (preoperational chemotherapy[Title/Abstract])) OR (preoperatory chemotherapy[Title/Abstract])) OR (presurgery chemotherapy[Title/Abstract])) OR (presurgical chemotherapy[Title/Abstract]) |
|  | #10 | #8 OR #9 |
| **P** AND **I** | #11 | #7 AND #10 |

**Note**: we performed the "P AND I" search strategies (without C, O, S) for improving the recall, although doing so will increase our workload of literature screening.
